# Ticking clocks and tamed transplants: how circadian timing rewrites graft-versus-host disease outcomes

**DOI:** 10.1038/s41392-025-02289-6

**Published:** 2025-06-25

**Authors:** Yishan Ye, He Huang, Mohamad Mohty

**Affiliations:** 1 Department of Hematology and Cell therapy, Hospital Saint-Antoine, Sorbonne University, Paris, France; 2 Bone Marrow Transplantation Center, The First Affiliated Hospital, Zhejiang University School of Medicine, Hangzhou, China

**Keywords:** Haematological cancer, Translational research

In a recent study published in *Cell*,^1^ Hou et al. presents a compelling investigation into the impact of circadian timing on the incidence and severity of acute graft-versus-host disease (aGVHD) following allogeneic hematopoietic stem cell transplantation (allo-HSCT). Through a combination of preclinical murine models and clinical cohort analyses, the authors demonstrate that the timing of stem cell infusion relative to the recipient’s circadian rhythm plays a pivotal role in modulating immune responses post-transplantation.

GVHD remains a major barrier to the success of allo-HSCT, contributing significantly to morbidity and mortality despite current prophylactic strategies.^2^ This study observed that stem cell infusion during the early part of the day (morning) leads to a significantly lower incidence and severity of aGVHD compared to afternoon or evening infusions. Mechanistically, the authors identify the inflammatory cytokine interleukin (IL)-1α as a key player mediating the circadian effects. Mice and patients infused during their circadian active phase (active phase in mice corresponds to the night; in humans corresponds to the morning) exhibit lower baseline inflammation and reduced donor T cell activation. Conversely, infusions performed during active circadian phases are associated with heightened inflammatory responses and more severe GVHD manifestations. Neutralizing IL-1α in murine models leads to a reduction in aGVHD severity, underscoring its causal role. Two clinical cohorts were analyzed: a retrospective single-center group (*n* = 204) and an external multicenter validation cohort (*n* = 543). Both cohorts confirmed that patients who received infusions before 2 p.m. had better GVHD outcomes and improved GVHD-free relapse-free survival. The circadian effects were consistently observed across different transplant centers and conditioning regimens, suggesting the broad applicability of this finding.

**Fig. 1:**
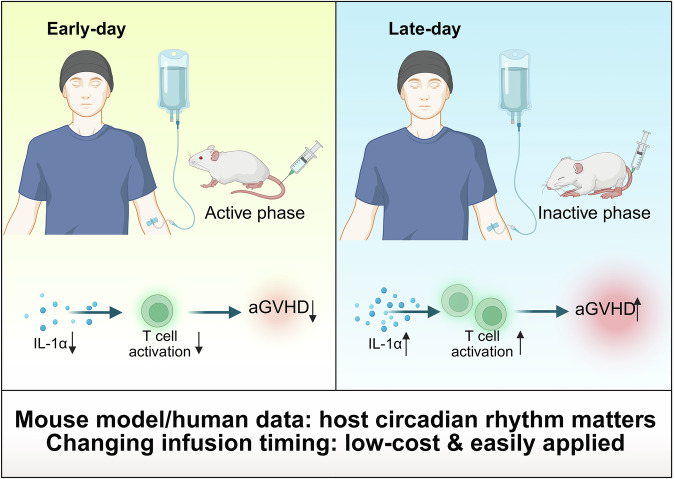
Stem cell infusion during the early part of the day (morning) leads to a significantly lower incidence and severity of aGVHD compared to afternoon or evening infusions. The inflammatory cytokine IL-1α plays a key role in circadian effects by mediating T cell activation. Changing infusion timing is a relatively low-cost, easily implementable intervention in clinical practice. The figure is created with Biorender.com

One of the most significant strengths of this study is its integrated translational approach, linking robust murine experiments with comprehensive human clinical data. This dual methodology supports the central hypothesis and strengthens the credibility of the findings. The large patient cohorts, including an external multicenter validation set, add substantial statistical power and generalizability. By replicating findings across different hospitals and transplant conditions, the authors reduce potential center-specific confounding. From a mechanistic standpoint, the identification of IL-1α as a circadian-regulated cytokine with a causal role in T cell activation and GVHD pathogenesis is highly valuable. The authors not only establish a correlation but also intervene using IL-1α neutralizing antibodies in vivo, providing direct evidence of causality. Another strength is the rigorous control of experimental variables in the murine studies. The use of Per1/2 knockout mice and suprachiasmatic nucleus lesions demonstrates the role of the endogenous circadian clock, rather than external light cues.^3^ Furthermore, reciprocal transplants between donors and recipients with different circadian timings clearly establish that the recipient’s rhythm, not the donor’s, is the critical determinant. The study also opens an avenue for clinical translation without the need for new drugs. Changing infusion timing is a relatively low-cost, easily implementable intervention that could have a profound impact on patient outcomes. The scientific significance and translational value of this study are illustrated in Fig. 1.

Despite its strengths, the study is not without limitations. First, the retrospective nature of the clinical analysis limits the ability to establish definitive causality in humans. Although multivariate analyses accounted for many confounders, unmeasured variables (e.g., staffing patterns, infusion rates, real-time inflammatory markers) could influence outcomes. Second, while the authors provide extensive murine data, not all patients underwent identical conditioning or GVHD prophylaxis. The heterogeneity in clinical protocols, though partially addressed in multivariate regression models, still introduces variability. Third, the cut-off point of 2 p.m., while biologically plausible, is somewhat arbitrary. Though sensitivity analyses were performed with varying cut-offs, future prospective studies are needed to optimize this timing and investigate whether earlier or later thresholds might yield even better outcomes. Fourth, the focus on peripheral blood stem cell transplants with fresh grafts excludes other graft sources, such as bone marrow or umbilical cord blood, and cryopreserved products. This is particularly relevant in the context of centers where cryopreserved grafts/bone marrow/umbilical cord blood are frequently used due to logistical constraints. Moreover, the timing of fresh peripheral blood stem cell infusion may be influenced by factors such as donor age, body weight, and collection efficiency. Additional validation using other graft types or cryopreserved grafts will help us conclude whether circadian modulation applies equally across all graft types and preservation conditions. In addition, although IL-1α is identified as a key mediator, other cytokines and pathways likely contribute to the circadian influence on immune reactivity.^4^ The complex interplay between various inflammatory and regulatory signals warrants deeper exploration. Lastly, the entire clinical dataset was obtained from Chinese transplant centers, which might limit the generalizability to other global populations with different genetic, environmental, and medical practice factors.^5^

The implications of this study are potentially transformative. The finding that something as simple as changing the timing of stem cell infusion can significantly reduce aGVHD opens the door to a novel, non-pharmacologic strategy in transplantation medicine. Given the ease of implementation, this approach could rapidly become standard practice, pending validation in prospective, randomized controlled trials, including harmonization of infusion protocols and timing across centers. Future research should explore the optimal time window for stem cell infusion, potentially tailoring it to individual patients based on chronotype, metabolic profile, or pre-infusion cytokine levels. In addition, studies should determine whether circadian timing affects other critical endpoints, such as chronic GVHD, relapse, immune reconstitution, and infection rates. The role of IL-1α as a therapeutic target also deserves further attention. Timing prophylactic administration of IL-1α antagonists, or other immunomodulators, could offer synergistic benefits with early-day infusions, particularly in high-risk patients. Beyond hematopoietic transplantation, the findings raise broader questions about the role of circadian biology in immunotherapy. As immunotherapies gain traction in oncology and other fields, understanding the circadian modulation of immune activation could fine-tune therapeutic windows and improve outcomes. Finally, this work highlights the need for greater integration of chronobiology into clinical trial design and healthcare delivery. Much like how dosing and drug selection are tailored to patient characteristics, the time of day may become an essential parameter in personalized medicine.

Hou et al.‘s study adds a novel, impactful dimension to our understanding of transplant immunology. By revealing the critical role of circadian timing in immune modulation and aGVHD risk, it provides a promising, practical strategy to improve outcomes after allo-HSCT. As the field moves forward, the integration of circadian biology may become a routine component of clinical practice, not only in transplantation but also across a wide array of immune-related disorders.
